# Stigmatization of schizophrenic individuals and its correlation to the fear of violent offence. Should we be concerned?

**DOI:** 10.1016/j.amsu.2022.104666

**Published:** 2022-09-19

**Authors:** Mariam Adil, Isha Atiq, Aayat Ellahi

**Affiliations:** a Department of Internal Medicine, Dow University of Health Sciences, Karachi, Pakistan; b Department of Internal Medicine, Jinnah Sindh Medical University, Karachi, Pakistan

**Keywords:** Stigma, Schizophrenia, Violence, Etiology

Schizophrenia is one of the most critical and devitalizing psychiatric conditions worldwide. Those diagnosed with this mental health disorder often experience symptoms such as hallucinations, lack of motivation and delusions [1]. These psychosocial deficiencies and cognitive impairment often lead to a range of negative stereotypes to be associated with this highly stigmatized neuropsychiatric condition. Existing stereotypes and the reluctance to interact with schizophrenic individuals has been correlated to the fear of violent offence [2]. The association between schizophrenia and the incidence of violence has been investigated to ascertain whether this fear is warranted [3].

For instance, a study found pronounced aggression among offenders with schizophrenia (68%), as compared to offenders without a mental illness (38%) [3]. Another study reported that violence (17.25%), running away and suicide attempts commonly co-occurred among schizophrenic patients [4]. This rather strengthens the belief that schizophrenia itself may be the sole cause of violent behavior. In 2021, Whiting et al. found an exacerbated risk of violent outcomes among individuals with schizophrenia spectrum disorders (SSD) compared with community controls. However, according to this report, the overall risk of violence remains low (less than 1 in 20 in SSD women, and less than 1 in 4 for SSD men over a 35-year period for violent arrests and crimes) [5].

Furthermore, analyses have highlighted that external factors, such as drug abuse, income, immigration, and marital status may significantly contribute to the development of violent behavior in schizophrenic patients. This shows that this condition may not be the mere reason of violence. Unfortunately, schizophrenia spectrum disorders are often misunderstood, and thus schizophrenic patients are frequently victimized. Approximately 64.5% of 5871 schizophrenic individuals experienced stigma in a study conducted by Gerlinger et al. [6] 1054 schizophrenic patients included in another study had committed 1 or more violent offence and 27.6% of who had committed an offence had existing substance abuse problems [2]. Due to the scarcity of the knowledge regarding the cause of aggression and violence among these individuals, there is debate surrounding the course of treatment to be followed. Apprehending the pathways to aggression is essential for designing more efficacious treatment trials in the future [7]. A recent study has also observed the role of regional factors on violence to others, suggesting the need for multi-sectorial targeted control strategies [8]. If etiological factors were to be investigated and the aforementioned problems were to be addressed, violent tendencies amongst these individuals may be curtailed.

Stigmatization of schizophrenic individuals may have severe consequences. The mentally challenged individuals experiencing disrepute may find it difficult to recover from their pre-existing symptoms and have a low quality of life due to their inability to integrate in the community [9]. While the treatment of schizophrenics may be affected by this social issue, it may also hinder the ability of some psychiatrists to deliver a diagnosis of schizophrenia. A study conducted by Clark et al. and Rowe et al. found that their results suggested that psychiatrists recognize people with schizophrenia as being more vulnerable to performing violent attacks than those with bipolar affective disorder, and this preconception leads to a diagnostic bias. This bias in diagnosis may concurrently affect the management of the patient and aggravate the stigma associated with a diagnosis of schizophrenia.

There is thus a growing need for health care professionals and the general population to address the stigmatization of these individuals and explore the extent of contribution of other factors. It is important to educate the public regarding the accurate association between schizophrenia and related factors. Mental health specialists and the media can help spread awareness among individuals about the negative impact of social isolation of schizophrenic patients by discussing the current social environment for SSD patients in interactive television programs. Moreover, innovative schemes can be introduced to allow interaction between these individuals and the general population to strengthen communication and trust. 10.13039/100014337Furthermore, schizophrenic patients can be more keenly treated for underlying psychiatric conditions and more funding can be generated and allocated to their therapy. Taking all of these necessary measures will help rectify the mentality of the public and allow adequate treatment regimens, resulting in an improved quality of life of schizophrenic individuals [10].

## Ethical approval

N/A.

## Sources of funding

None.

## Author statement

Mariam Adil proposed the idea.

Mariam Adil, Isha Atiq collected the data and completed the manuscript.

Aayat Ellahi finalized the manuscript did the proof-reading and submission of article. All authors approved the final version of article.

## Consent

N/A.

## Registration of research studies

Name of the registry: N/A.

Unique Identifying number or registration ID: N/A.

Hyperlink to your specific registration (must be publicly accessible and will be checked): N/A.

## Guarantor

Mariam Adil.

Isha Atiq.

Aayat Ellahi.

## Declaration of competing interest

None.
